# Medical-Grade Honey as an Alternative Treatment for Antibiotics in Non-Healing Wounds—A Prospective Case Series

**DOI:** 10.3390/antibiotics10080918

**Published:** 2021-07-28

**Authors:** Adéla Holubová, Lucie Chlupáčová, Lada Cetlová, Niels A. J. Cremers, Andrea Pokorná

**Affiliations:** 1 Faculty of Health and Social Sciences, University of Bohemia, 370 11 České Budějovice, Czech Republic; 2 DiaPodi Care spol. s r.o., 392 01 Soběslav, Czech Republic; diapodicare@seznam.cz; 3 Department of Health Sciences, College of Polytechnics Jihlava, 586 01 Jihlava, Czech Republic; lada.cetlova@vspj.cz (L.C.); apokorna@med.muni.cz (A.P.); 4 Triticum Exploitatie BV, 6222 NK Maastricht, The Netherlands; niels@mesitran.com; 5 Department of Nursing and Midwifery, Faculty of Medicine, Masaryk University, 625 00 Brno, Czech Republic

**Keywords:** medical grade honey, antibiotic replacement, infections, wounds, objective wound assessment

## Abstract

Non-healing wounds are usually colonised by various types of bacteria. An alternative to antibiotic treatment in patients with infected wounds with local signs of inflammation may be medical-grade honey (MGH), which favourably affects the healing process with its antimicrobial, antioxidant, anti-inflammatory, and immunomodulatory properties. The objective of this study was to evaluate the effect of MGH therapy on the healing process of non-healing wounds of various aetiologies and different wound colonisations. Prospective, observation–intervention case studies (*n* = 9) of patients with wounds of various aetiologies (venous leg ulcers, diabetic foot ulcers, surgical wound dehiscence) are presented. All wounds were treated with MGH and the healing trajectory was rigorously and objectively monitored. In all cases, pain, odour, and exudation were quickly resolved, which led to an improvement in the quality of life of patients. Despite the proven bacterial microflora in wounds, antibiotic treatment was not necessary. The effects of MGH alleviated the signs of local infection until their complete elimination. In eight out of nine cases, the non-healing wound was completely healed. MGH has antimicrobial, anti-inflammatory, and antioxidant effects in wounds of various aetiologies and forms an effective alternative for the use of antibiotics for treating locally infected wounds.

## 1. Introduction

The Wound Healing Society has defined four categories of non-healing ulcers based on their aetiology: pressure ulcers, diabetic ulcers, and ulcers due to venous insufficiency or arterial insufficiency [1]. A non-healing wound, also called a hard to heal or chronic wound, is damage to the skin that heals unusually slow. Depending on the definition, a non-healing wound is usually present for at least 4 weeks [2]. Non-healing wounds have a major impact on the patient’s quality of life [3,4]. Therefore, the main goal in the treatment of non-healing wounds is not only their healing but also the alleviation of unpleasant wound symptoms, such as pain and malodour [5]. An objective assessment of non-healing wounds, including the overall condition of the patient, is very important to develop a realistic treatment plan and to determine cost-effectivity [6,7,8,9].

The wound healing process is complex and dynamic [10,11]. Non-healing wounds are often critically colonized by various types of microorganisms. Local signs of inflammation in the wound include redness, oedema, increase in local temperature, tissue damage, and pain. In difficult-to-heal or non-healing wounds, they also include prolonged healing, the presence of abnormal granulation tissue, increase in wound size, and increased amount of exudate [12]. Antibiotic treatment based only on the presence of bacteria is not a good practice and is potentially dangerous with respect to the formation of polyresistant microbial strains, and ultimately leads to increased therapy costs and resource wastage [13,14]. The amount and behaviour of bacteria in wounds varies from contamination to invasive infection. Biofilm is often present, which is a bacterial colony surrounded by an extracellular matrix consisting of polysaccharides forming a shield to antimicrobial agents and increasing resistance [15]. One of the options to reduce the bioburden of the wound bed is sharp debridement. Debridement refers to a process in which avital tissue, coatings, microbial load (including biofilms), and tissue debris are removed from a wound [16,17,18]. Sharp debridement accelerates wound cleansing and shortens the total time required for tissue repair, but has low selectivity and often damages vital tissues [19,20]. Therefore, it is appropriate to use less invasive but similarly effective autolytic debridement techniques. One of them is the use of medical-grade honey (MGH).

Honey has been used to treat wounds and local infections for more than 5000 years [21]. MGH is carefully selected and gamma-sterilized in order to ensure safe use for wound care [22,23]. MGH has a positive effect on the healing process with its antimicrobial, antioxidant, anti-inflammatory, and immunomodulatory properties. It also stimulates the production of hydrogen peroxide at a concentration that is not toxic to damaged tissues [24,25]. It also supports the activity of the immune system, debridement, and stimulates regenerative processes in the wound [26]. MGH decreases wound healing time and is cost-effective [27]. The aim of our study was to assess the effect of MGH on the healing of non-healing wounds. In this prospective case series, nine patients with infected non-healing wounds were treated with MGH in the absence of antibiotics. Our hypothesis was that MGH could replace the use of antibiotics in locally infected wounds and promote debridement, wound healing, and patients’ quality of life.

## 2. Results

### 2.1. Case 1: Dehisced Surgical Wound

**Figure 1:**
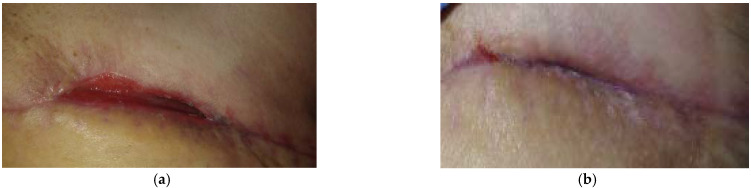
Case 1: Dehiscence of the surgical wound on the left breast. (**a**) Local finding at initial examination, day 0 (start of MGH treatment). (**b**) Complete wound healing on follow-up examination at day 35.

A 57-year-old female patient presented with a dehisced surgical wound at her left breast following a breast-conserving operation as a result of being diagnosed with breast cancer (Figure 1a). Relevant comorbidities included ovarian cancer and having diabetes mellitus (DM) type 2, which could subsequently affect the healing. Previous treatments with sterile bioceramic dressings for three months were ineffective. The wound dimensions upon presentation were 6 cm in length, 1.5 cm in width, and ranging from 0.5 cm to 2 cm in depth (deeper towards axilla). The wound consisted mainly (roughly 95%) of granulation tissue and 5% slough. Low levels of exudate (thin, water-like) were produced. Local signs of infection included delayed healing, pain, and redness. The pain was scored on a visual analogue (VAS)-scale by the patient for pain level during the daytime and during wound care treatment. Pain level was 5 during the daytime and 8 during treatment. L-Mesitran^®^ Soft gel (MGH) was applied inside the lesion and followed by L-Mesitran^®^ Tulle (MGH) to ensure contact to the wound. Mepilex foam (foam dressing) was applied as a secondary dressing. Wound dressings were performed by the patient at home at 72 h intervals for the first two weeks. Pain and redness disappeared after 14 days of treatment. Due to the positive evolution of the healing, the dressing changes interval were extended to every four days. The wound was completely healed after 35 days of MGH treatment without complications (Figure 1b).

### 2.2. Case 2: Venous Leg Ulcer

**Figure 2:**
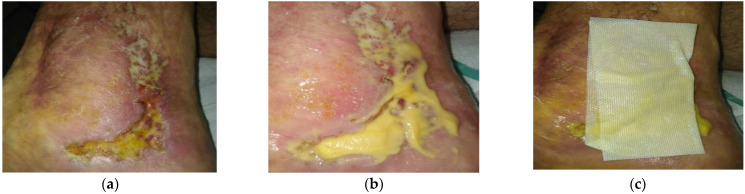

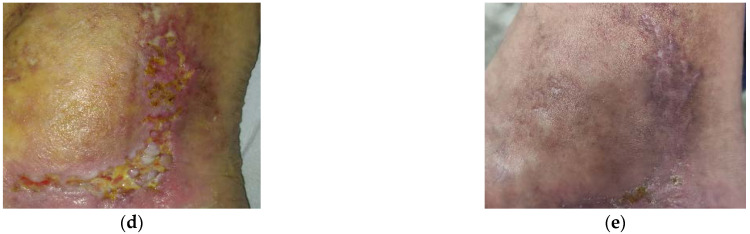
Case 2: Venous leg ulcer on the right lower leg. (**a**) Local finding at initial examination, day 0 (start of MGH treatment). (**b**) Example of L-Mesitran^®^ Ointment application. (**c**) Example of L-Mesitran^®^ Tulle application. (**d**) Follow-up examination at day 20. (**e**) Complete wound healing on follow-up examination at day 67.

A 43-year-old male patient presented with a venous leg ulcer at his right lower leg (Figure 2a). Relevant comorbidities included chronic venous insufficiency (CHVI), diabetes mellitus (DM), obesity (BMI 30), and repeated venous lower ulcers. Previous treatments with iodinated povidone solution for two months were ineffective. The wound dimensions upon presentation were 6 cm in length, 5 cm in width, and 1 cm in depth. The wound consisted of 5% of granulation tissue and 95% slough. Low levels of exudate (thin, water-like) were produced. Local signs of infection included pain, erythema, local warmth, exudate, delayed healing, and malodour. A microbiological swab was performed, in which *Enterococcus faecalis* (resistant to Trimethoprim + sulphonamide, neomycin, clindamycin, gentamicin; and sensitive to ampicillin, nitrofurantoin, norfloxacin, bacitracin, ciprofloxacin, and chloramphenicol) and *Escherichia coli* without resistance were detected (sensitive to ampicillin, aminopenicillin, cefuroxime, trimethoprim + sulphonamide, cefpodoxime, neomycin, gentamicin, ciprofloxacin, and chloramphenicol). Pain level was 8 during the daytime and 9 during treatment. L-Mesitran^®^ Ointment (MGH) was applied to the wound (Figure 2b) and followed by L-Mesitran^®^ Tulle (Figure 2c) to ensure contact to the wound bed. Suprasorb P foam (foam dressing) was applied as a secondary dressing. Wound dressings were performed by the patient at home at 48 h intervals for the first two weeks. After 20 days, the wound dimensions upon presentation were 4 cm in length, 4 cm in width, and 0.5 cm in depth (Figure 2d). The wound consisted of 20% of granulation tissue, 30% epithelializing, and 50% slough, and malodour and infection disappeared. Pain levels gradually decreased and after 20 days of treatment, the pain level was 2 (VAS). Due to the positive development of healing, the dressing changes were extended to every three days. The wound was completely healed after 67 days of MGH treatment without complications (Figure 2e).

### 2.3. Case 3: Venous Leg Ulcer

**Figure 3:**
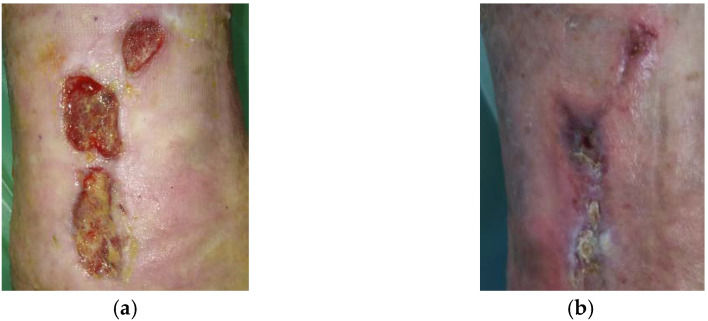
Case 3: Venous leg ulcer on the right lower leg. (**a**) Local finding at initial examination, day 0 (start of MGH treatment). (**b**) Complete wound healing on follow-up examination at day 79.

A 72-year-old male patient presented with a venous leg ulcer on his right lower leg (Figure 3a). Relevant comorbidities included CHVI, DM, hypertension (HT), and a medical history of thrombosis of the right lower leg without acute symptomatology. Previous treatments with iodinated povidone solution for six weeks were ineffective. Upon presentation, the wound dimensions were 14 cm in length, 4 cm in width, and 1 cm in depth. The wound consisted of 30% of granulation tissue and 70% slough. Medium levels of exudate (thin, water-like) were produced. Local signs of infection included pain, delayed healing, and malodour. A swab was performed in which *Enterococcus faecalis* (resistant to trimethoprim + sulphonamide, neomycin, clindamycin, and gentamicin; and sensitive to ampicillin, nitrofurantoin, bacitracin, ciprofloxacin, chloramphenicol) and *Escherichia coli* without resistance were detected (and sensitive to ampicillin, aminopenicillin, cefuroxime, trimethoprim + sulphonamide, cefpodoxime, gentamicin, ciprofloxacin, and chloramphenicol). Pain level was 6 during the daytime and 9 during treatment. L-Mesitran^®^ Ointment was applied to the wound and followed by L-Mesitran^®^ Tulle. Resposorb^®^ Super (super absorbent dressing) was applied as a secondary dressing. Wound dressings were performed by the patient at home at 48 h intervals for the first two weeks. Pain levels gradually decreased and after 14 days of treatment, the pain was tolerated at pain level 1 (daytime) and 2 (during treatment–procedural pain). Due to the positive development of healing, the dressing changes were extended to every three days. After 42 days, only L-Mesitran^®^ Tulle was applied to the wound. Suprasorb P foam was applied as a secondary dressing. The wound was completely healed after 79 days of MGH treatment without complications (Figure 3b).

### 2.4. Case 4: Venous Leg Ulcer

**Figure 4:**
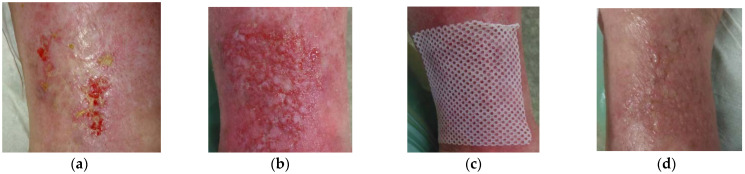
Case 4: Venous leg ulcer on the left lower leg. (**a**) Local finding at initial examination, day 0 (start of MGH treatment). (**b**) Follow-up examination at day 51. (**c**) Example of L-Mesitran^®^ Net application. (**d**) Complete wound healing on follow-up examination at day 62.

A 75-year-old female patient presented with a venous leg ulcer on her left lower leg (Figure 4a). Relevant comorbidities included CHVI, DM, HT, and the patient underwent varices surgery on the left lower leg in the past with no actual symptomatology. Previous treatments with antiseptic dressing with silver nanoparticles for two months were ineffective. Upon presentation, the wound dimensions were 1.5 cm in length, 1.5 cm in width, and 0.8 cm in depth (top wound) and 3 cm in length, 2 cm in width, and 0.8 cm in depth (lower wound), both in craniocaudal direction. The wound bed consisted of 30% of granulation tissue and 70% slough. Medium levels of exudate (thin, water-like) were produced. Local signs of infection included pain and delayed healing. A wound swab confirmed the presence of *Staphylococcus aureus* resistant to clindamycin and sensitive to trimethoprim + sulphonamide, norfloxacin, neomycin, bacitracin, chloramphenicol, and gentamicin. Pain level was 5 during the daytime and 7 during treatment (procedural pain). L-Mesitran^®^ Ointment was applied to the wound and followed by L-Mesitran^®^ Tulle. Resposorb^®^ Super (foam dressing) was applied as a secondary dressing. Wound dressings were performed by the patient at home at 48 h intervals for the first two weeks. After 51 days, the wound dimensions were 6 cm in length, 6 cm in width, and 0.5 cm in depth (Figure 4b). The wound bed consisted of 70% of granulation tissue and 30% epithelialization tissue. Pain levels gradually decreased and after 14 days of treatment, the pain level during the daytime was 1 and during treatment it was rated at level 2. After the proposed topical treatment, the wound bed was cleansed, the granulation and epithelialization phases took place, but the wound area increased despite getting more superficial. There was also a reduction in wound exudate and pain, and local signs of infection disappeared. Due to these aspects, the dressing was changed to L-Mesitran^®^ Net (MGH) (Figure 4c) and PermaFoam^®^ classic (foam dressing) was used as a secondary dressing. Due to the positive development of healing, the dressing changes were extended to every three days. The wound was completely healed after 62 days of MGH treatment without complications (Figure 4d).

### 2.5. Case 5: Diabetic Foot Ulcer

**Figure 5:**
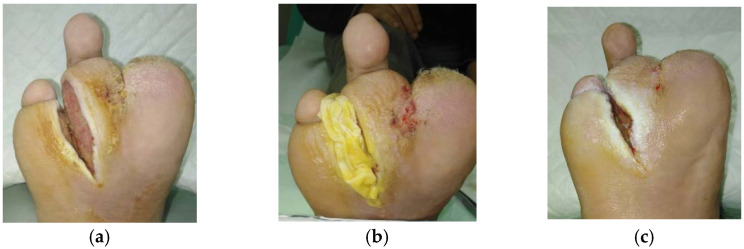
Case 5: Diabetic foot ulcer on the right foot. (**a**) Local finding at initial examination, day 0 (start of MGH treatment). (**b**) Example of L-Mesitran^®^ Tulle application. (**c**) Improved wound healing on follow-up examination at day 25.

A 59-year-old male patient presented with a diabetic foot ulcer at his right foot (Figure 5a). Relevant comorbidities included repeated diabetic gangrene, repeated amputation of toes on the right foot, diabetic neuropathy, DM, HT, and obesity (BMI 32)**.** Previous treatments with iodinated povidone solution for six weeks were ineffective. Upon presentation, the wound dimensions were 8 cm in length, 3 cm in width, and 5 cm in depth. The wound consisted of 80% of granulation tissue and 20% slough. High levels of exudate (thin, water-like) were produced. Local signs of infection included low level of neuropathic pain, exudate, delayed healing, and malodour. There was maceration, hyperkeratosis and callus in the peri-wound skin. A wound swab confirmed the presence of *Proteus mirabilis* (resistant to ampicillin, aminopenicillin, cefuroxime, trimethoprim + sulphonamide, cefpodoxime, gentamicin, ciprofloxacin, chloramphenicol, and cefotaxime; and sensitive to neomycin, amikacin, ceftazidime, ertapenem, meropenem), *Staphylococcus aureus* (resistant to clindamycin, gentamicin; and sensitive to cefoxitin, trimethoprim + sulphonamide, norfloxacin, neomycin, bacitracin, chloramphenicol, fusidic acid, and mupirocin) and *Acinetobacter baumanii* (resistant to ampicillin, aminopenicillin, cefuroxime, cefpodoxime, chloramphenicol, cefotaxime; and sensitive to trimethoprim + sulphonamide, neomycin, gentamicin, ciprofloxacin, amikacin, ceftazidime). Pain level was 1 during the daytime as well as during treatment (diabetic neuropathy). L-Mesitran^®^ Ointment was applied to the wound, followed by L-Mesitran^®^ Tulle (Figure 5b) and Resposorb^®^ Super as a secondary dressing. Wound dressings were performed by the patient at home at 48 h intervals due to bacterial findings and heavy wound exudation for first follow-up examination. On day 25, the wound dimensions were 5 cm in length, 1.5 cm in width, and 1 cm in depth (Figure 5c). The wound consisted of 90% of granulation tissue and 10% slough. Pain and odour levels gradually decreased over time and were completely absent on day 25. After the proposed topical treatment, the wound area was reduced and the wound bed was cleaned. Due to the occurrence of maceration, likely due to non-compliance to the treatment, topical treatment was slightly adjusted. The application of L-Mesitran^®^ Ointment was omitted and the patient was advised to continue treatment at home at 48 h intervals due to moderate wound exudation.

### 2.6. Case 6: Venous Leg Ulcer

**Figure 6:**
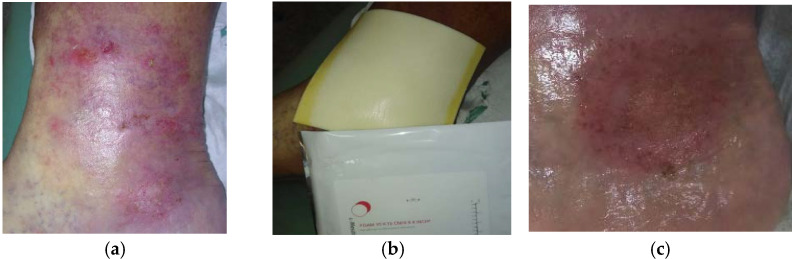
Case 6: Venous leg ulcer on the right lower leg. (**a**) Local finding at initial examination, day 0 (start of MGH treatment). (**b**) Example of L-Mesitran^®^ Foam application. (**c**) Complete wound healing on follow-up examination at day 15.

A 54-year-old male patient presented with a venous leg ulcer at his right lower leg (Figure 6a). Relevant comorbidities included CHVI, HT, DM, hyperlipidaemia, and hyperuricemia. Previous treatments with iodinated povidone solution for seven weeks were ineffective. Upon presentation, the wound dimensions were 6 cm in length, 6 cm in width, and 0.5 cm in depth. The wound consisted of 95% of granulation tissue and 5% slough. Low levels of exudate (thin, water-like) were produced. Local signs of infection included pain and delayed healing. A wound swab confirmed the presence of *Enterococcus faecalis* (resistant to trimethoprim + sulphonamide, neomycin, clindamycin, gentamicin; and sensitive to ampicillin, nitrofurantoin, ciprofloxacin, and chloramphenicol). Pain level was 6 during the daytime and 8 during treatment. L-Mesitran^®^ Foam was applied to the wound (Figure 6b). Wound dressings were performed by the patient at home at 72 h intervals. Pain levels gradually decreased over time and after 10 days of treatment, the pain level during daytime was 0 and 1 during treatment. The wound was completely healed after 15 days of MGH treatment without complications (Figure 6c).

### 2.7. Case 7: Venous Leg Ulcer

**Figure 7:**
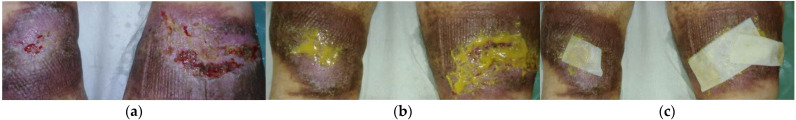

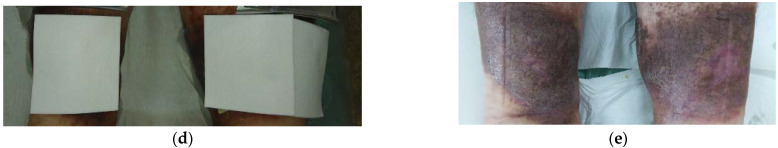
Case 7: Bilateral venous leg ulcers. (**a**) Local finding at initial examination, day 0 (start of MGH treatment). (**b**) Example of L-Mesitran^®^ Ointment application. (**c**) Example of L-Mesitran^®^ Tulle application. (**d**) Example of Vacutex^®^ application. (**e**) Complete wound healing on follow-up examination at day 54.

A 52-year-old male patient presented with bilateral venous leg ulcers (Figure 7a). Relevant comorbidities included CHVI, DM, HT hyperlipidaemia, hyperuricemia, morbid obesity (BMI 45), and repeated venous leg ulcers. Previous treatments with iodinated povidone solution for two months were ineffective. Upon presentation, the wound dimensions on the right leg were 2 cm in length, 2 cm in width, and 1 cm in depth. On the left leg, the wound dimensions were 10 cm in length, 12 cm in width, and 1 cm in depth. The wound bed consisted of 80% of granulation tissue and 20% slough. Medium levels of exudate (thin, water-like) were produced. Local signs of infection included pain and delayed healing. A wound swab confirmed the presence of *Proteus mirabilis* (resistant to ampicillin, aminopenicillin, cefuroxime, trimethoprim + sulphonamide, cefpodoxime, gentamicin, ciprofloxacin, and cefotaxime and sensitive to neomycin, amikacin, ceftazidime, meropenem). *Staphylococcus aureus* (resistant to clindamycin; and sensitive to trimethoprim + sulphonamide, norfloxacin, neomycin, bacitracin, and chloramphenicol). Pain level was 5 during daytime and 8 during treatment. L-Mesitran^®^ Ointment was applied to the wound (Figure 7b) and followed by L-Mesitran^®^ Tulle (Figure 7c) to ensure contact to the wound. Vacutex^®^ (capillary action dressing) was applied as a secondary dressing (Figure 7d). Dressing changes were performed by the patient at home at 48 h intervals because of the heavy bacterial colonization and moderate wound exudation. Pain levels gradually decreased and after 14 days of treatment, the pain level was 1 during the daytime and 2 during treatment. After 14 days of treatment, the interval of dressing changes was prolonged to 72 h. Since the wound became more superficial, L-Mesitran^®^ Ointment was omitted from the treatment and L-Mesitran^®^ Tulle was applied and PermaFoam^®^ classic was used as a secondary dressing. The wound was completely healed after 54 days of MGH treatment without complications (Figure 7e).

### 2.8. Case 8: Diabetic Foot Ulcer

**Figure 8:**
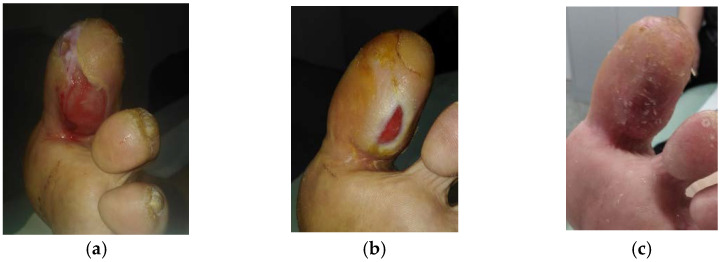
Case 8: Diabetic foot ulcer on the left foot. (**a**) Local finding at initial examination, day 0 (start of MGH treatment). (**b**) Follow-up examination at day 21. (**c**) Complete wound healing on follow-up examination at day 44.

A 51-year-old female patient presented with a diabetic foot ulcer on her left foot (Figure 8a). Relevant comorbidities included DM, HT, morbid obesity (BMI 45), and repeated wounds on the right foot due to diabetic foot syndrome. Previous treatments with iodinated povidone solution for two months were ineffective. Upon presentation, the wound dimensions were 5 cm in length, 3 cm in width, and 1 cm in depth. The wound consisted of 90% of granulation tissue, 5% slough, and 5% epithelialization tissue. Low levels of exudate (thin, water-like) were produced. Local signs of infection included pain and delayed healing. A wound swab confirmed the presence of *Proteus mirabilis* (resistant to ampicillin, aminopenicillin, cefuroxime, trimethoprim + sulphonamide, cefpodoxime, gentamicin ciprofloxacin, chloramphenicol, cefotaxime; and sensitive to neomycin, amikacin, ceftazidime, ertapenem, and meropenem). *Staphylococcus aureus* (resistant to clindamycin, gentamicin; and sensitive to trimethoprim + sulphonamide, norfloxacin, neomycin, bacitracin, chloramphenicol, fusidic acid, mupirocin) and *Enterococcus faecalis* (resistant to trimethoprim + sulphonamide, neomycin, clindamycin, gentamicin; and sensitive to ampicillin, nitrofurantoin, norfloxacin, bacitracin, ciprofloxacin, and chloramphenicol). Pain level was 5 during the daytime and 8 during treatment. L-Mesitran^®^ Ointment was applied to the wound and L-Mesitran^®^ Foam was applied as a secondary dressing. Dressing changes were performed by the patient at home at 48 h intervals due to bacterial findings. On day 21, the wound dimensions upon presentation were 1.5 cm in length, 0.5 cm in width, and 0.5 cm in depth (Figure 8b). Pain levels gradually decreased and after 21 days of treatment, the pain level was 1 during the daytime and during treatment. The interval of the dressing changes was prolonged to 72 h and continued with applying only L-Mesitran^®^ Foam. The wound was completely healed after 44 days of MGH treatment without complications (Figure 8c).

### 2.9. Case 9: Venous Leg Ulcer

**Figure 9:**
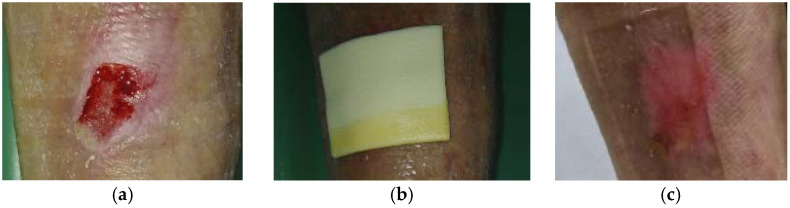
Case 9: Venous leg ulcer on the right lower leg. (**a**) Local finding at initial examination, day 0 (start of MGH treatment). (**b**) Example of L-Mesitran^®^ Foam application. (**c**) Complete wound healing on follow-up examination at day 17.

A 49-year-old male patient presented with a venous leg ulcer at his right lower leg (Figure 9a). Relevant comorbidities included DM, HT, hyperlipidaemia, and a medical history of thrombosis of the right lower leg without acute signs of ischemia. Previous treatments with antiseptic dressing with silver nanoparticles for seven weeks were ineffective. Upon presentation, the wound dimensions were 5 cm in length, 3 cm in width, and 1.5 cm in depth. The wound consisted of 90% of granulation tissue, 5% slough, and 5% epithelialization tissue. Low levels of exudate (thin, water-like) were produced. Local signs of infection included pain, malodour, and delayed healing. A wound swab confirmed the presence of *Enterococcus faecalis* (resistant to trimethoprim + sulphonamide, neomycin, clindamycin, gentamicin; and sensitive to ampicillin, nitrofurantoin, bacitracin, ciprofloxacin, chloramphenicol). Pain level was 5 during the daytime and 8 during treatment. L-Mesitran^®^ Soft (MGH) was applied to the wound and L-Mesitran^®^ Foam (Figure 9b) was applied as a secondary dressing. Dressing changes were performed by the patient at home at 48 h intervals. Pain and malodour levels gradually decreased and after 14 days of treatment, the pain was absent during the daytime (VAS score of 0) and at level 1 during treatment. Only L-Mesitran^®^ Foam was applied. The wound was completely healed after 17 days of MGH treatment without complications (Figure 9c).

## 3. Discussion

In all presented cases, the healing process was positively affected; the wound bed was cleansed; and the pain, odour, and exudation were completely eliminated. Despite the proven microbial burden in the wound by means of swab and cell culture examination, antibiotic treatment was not initiated and treated locally with only MGH. Signs of local infection were eliminated by the effect of MGH in the applied materials. In eight out of nine cases, the non-healing wound was completely healed. In one case (case 5) treatment with MGH continues. However, even in this case, there was a significant improvement in the local finding, but due to the low patient compliance, we have no further information. After proper training, the patients were able to perform the wound dressings themselves or with the help of family members at home, and they all considered it easy. No adverse effects of the applied topical material with MGH were observed.

The costs of wound management in developed countries are around 1–4% of total health care expenditure [15,28]. The number of non-healing wounds is expected to increase, due to longevity and comorbidities, such as obesity and diabetes mellitus, and hence, elevate economic impact [2,29,30]. MGH enables a faster healing process and can resolve local infections without the need for antibiotic treatment, and thus forms a potent alternative treatment.

A total of nine patients with wounds of various aetiologies were included in the study. MGH promoted autolytic debridement, led to the cleansing of the wound bed, and induced granulation tissue formation and epithelialization. This is in line with previous studies [14,31]. Debridement and cleansing can be attributed to the moist wound environment, its acidification, the osmotic activity, and oxygenation of the wound environment [14,31,32]. The prevalence of biofilms in non-healing wounds is estimated to be approximately 60% [33,34]. Biofilms have a high capacity for bacterial resistance and show increased resistance to host cellular responses and antiseptics [15,35]. MGH is effective in removing coating and necrosis from the wound bed, and thus may work in cases where antibiotics are ineffective [24]. Furthermore, MGH was also effective in eradicating MRSA in venous leg ulcers, so antibiotic-resistant strains can also be eliminated. The broad-spectrum antimicrobial activity of MGH on *Staphylococcus aureus*, *Pseudomonas aeruginosa,* and *Streptococcus* pathogens have been also confirmed by others [21,31,36]. Due to its antimicrobial mechanisms, including acidic pH, osmotic activity, and slow release of hydrogen peroxide, MGH is effective against a wide range of pathogens, including multi-resistant bacteria, fungi, and viruses [13,37,38]. Moreover, the use of MGH in wound management can reduce the use of antibiotics and topical antiseptics [32]. Repeated use of MGH materials is without risk of developing resistance [13,21]. In our study, all patients had local signs of infection and microbial burden in the wound bed was proven in eight out of nine patients (wound swab was not taken in one case). Antibiotic treatment was required in none of the cases. We have verified that topical treatment with MGH is a safe and easy-to-use alternative method for treating local infections. MGH has antimicrobial, antioxidant, and anti-inflammatory properties and is thus ideal for the treatment of infected wounds [14,39,40].

Alleviation of unpleasant symptoms that accompany non-healing wounds is important to improve the quality of a patient’s life [5]. Pain has not only a sensory component, but also an emotional component, which is associated with anxiety, depression, aggression, feelings of danger, helplessness, hopelessness, and loss of motivation [41,42]. Patients with wounds can also suffer from procedural pain [43]. Stress experienced during wound management increases cortisol levels, and this has a negative effect on wound healing [44,45]. In all patients, the pain was alleviated or eliminated after the first dressing change and the intensity of procedural pain was reduced. In all patients, the need for analgesic treatment was gradually reduced or eliminated. As supported by others, this can be attributed to the MGH that prevents incorporation of the wound dressing into the wound bed and subsequent damage of the new granulation during wound dressing [14,21].

Odour is another unpleasant symptom of infection in non-healing wounds. Wound odour has a negative effect on the patient’s psyche, is usually associated with abundant production of exudate, and the two factors can lead to social isolation of the patient [21]. In our study, a reduced odour and exudate was noticeable after the first dressing change (the shortest dressing interval was two days and odour and exudate disappeared after approximately 16 days of treatment). Only in patient number 5 was maceration of the wound edges (0.5 cm from the wound edge) observed. We believe that the patient applied a large amount of L-Mesitran^®^ Ointment to the wound and did not refresh the dressings frequently enough, which led to the accumulation of exudate in the secondary bandage and, subsequently, maceration. Therefore, it is always important to check exudate levels regularly and change dressings accordingly. After training, this issue was partially resolved, but there was low compliance in this patient. The high sugar content of MGH attracts lymph fluid and wound exudate out of the tissue and helps in the removal of exudate into the dressing [21]. This process, together with the anti-inflammatory activity of MGH, subsequently reduces oedema and pain [21]. Wound odour is produced by bacteria that metabolize serum, tissue proteins, and dead cells, leading to amino acid production and unpleasant odour [46,47]. Glucose in MGH acts as an alternative odourless substrate for these bacteria and thus eliminates odour [21,32,46,47]. In addition, the antimicrobial activity of honey will reduce the number of bacteria in the wound, thereby reducing odour [46,48]. This property is most evident within 24 h after the application of honey to the wound [49]. Also, in our study, patients reported a reduction in odour already after the first wound dressing (the shortest dressing interval was two days). Due to the beneficial healing, it was possible to extend the intervals between the individual dressings to up to four days. This of particular importance during the COVID-19 pandemic, when the availability of health services and personal contact with patients is really limited and often replaced by online or phone consultations. In order to get the best result, it is essential that the patients and their relatives cooperate with the treatment regimen and the hygiene measurements. Keeping them involved, maintaining regular appointments, and seeing progression in different aspects (odour, pain, wound progression) helps to keep the patients motivated. Non-healing wounds are an economic burden on healthcare systems. Extending the time between wound dressings, shortening the wound healing time, not administering antibiotics, and providing the ability to perform wound dressings at home (natural social environment or with the help of home care nurses) can significantly reduce the costs. MGH should be considered when non-healing wounds stay stagnant.

## 4. Materials and Methods

### Patient Selection

In a prospective observational study, MGH was applied to a selected group of patients with non-healing wounds. Inclusion criteria were having a non-healing wound with treatment lasting more than 6 weeks, type 2 diabetes mellitus, presence of local signs of inflammation, absence of systemic sings inflammation, pain including procedural pain, and patient agreement. Exclusion criteria were having an allergy to bee stings or MGH, systemic signs of inflammation, and patient disagreement.

A total of nine patients were included in the study, of which six were men and three women, the average age was 57 years (minimum 43 and maximum 75, with a median of 54 years). Six patients had leg ulcers, two had diabetic foot syndrome, and one had a dehisced surgical wound. Previous therapy consisted of iodine material in six cases, bioceramic dressing in one patient, and nano-silver material in two patients. All patients showed local signs of wound infection and signs of systemic infection were absent. The average assessment of pain intensity during the day reached 5.1 points and procedural pain averaged 7.1 points according to VAS. Most patients (*n* = 8) received oral analgesic treatment during the initial phase of wound treatment until pain levels were minimal—detailed descriptions about the pain level are part of the case summaries. In one patient, the pain was not pharmacologically treated, diabetic neuropathy was present.

Wound swabs were taken using the Levine technique upon first presentation to determine bacterial colonization [50]. Standard laboratory protocols and molecular testing using matrix-assisted laser desorption/ionization time of flight mass spectrometry (MALDI-ToF MS) were followed to identify the microorganisms and to determine their susceptibility and resistance profiles [51].

Wound characteristics and relevant patient information from the presented nine cases are summarized in Supplementary Table S1. Basic information about the patient, local assessment of the non-healing wound, pain, and the presence and cause of infection are available. Since all wounds were non-healing and locally infected, topical treatment with L-Mesitran was recommended.

## 5. Conclusions

In our prospective case series, we confirmed in a group of nine patients on an outpatient basis that MGH treatment has beneficial effects on the healing process of infected non-healing wounds of various aetiologies. The application of an MGH-containing dressing led to the activation of the healing process, stimulation of debridement, and a faster cleansing phase of the wound bed. MGH reduced odour and exudate secretion and maintained an optimal moist wound bed environment. Wound-related pain and procedural pain were significantly reduced and analgesia was reduced or stopped in all patients. Despite the local signs of infection and the presence of different microorganisms, MGH was effective to resolve infection, and thus replaced the need for antibiotics. Topical treatment of non-healing wounds with MGH dressing led to a lower frequency of wound dressings at home and lower financial costs of care. The healing and reduction in symptoms strongly improved the patients’ quality of life. MGH forms an attractive alternative to antibiotics to fight infections while enhancing the wound healing trajectory.

## Data Availability

The data that support the findings of this study are available from the corresponding author upon reasonable request. All data relevant to the study are included in the article. The data are safely stored as requested by Czech legislation in healthcare provider secured electronic system.

## Supplementary Materials

The following are available online at https://www.mdpi.com/article/10.3390/antibiotics10080918/s1. Table S1: Overview of the presented cases.
