# Supplementary material for: Medical-Grade Honey as an Alternative Treatment for Antibiotics in Non-Healing Wounds—A Prospective Case Series
Source: Antibiotics (Basel). 2021 Jul 28;10(8):918. doi: 10.3390/antibiotics10080918 (PMC8388796; doi:10.3390/antibiotics10080918)
Supplement: Supplementary file 1 [file antibiotics-10-00918-s001.zip › antibiotics-1293703-supplementary.pdf]

antibiotics

MDPI

Supplementary Table S1. Overview of the presented cases.

Case No.	Gender/ age (years)	Patient characteristics	Wound type and location	Previous treatments	Signs of infection	Bacterial determination	Pain VAS (score 1-10)	Antibiotic treatment
1	Female (57)	Ovarian and breast cancer, DM	Dehiscence of the surgical scar at left breast	Bioceramic sterile dressing applied for three months	Local: Delayed healing, pain	not performed	Daytime - 5 Procedural - 8	No
2	Male (43)	**CHVI, DM, obesity (BMI 30), repeated venous lower ulcers**	Venous leg ulcer at right lower leg	Iodinated povidone solution	Local: pain, erythema, local warmth, exudate, delayed healing, malodour	Enterococcus faecalis, Escherichia coli	Daytime - 8 Procedural - 9	No
3	Male (72)	**CHVI, DM, HT, condition after thrombosis of the right lower leg**	Venous leg ulcer at right lower leg	Iodinated povidone solution Antibiotic treatment – Ampicillin (tablets)	Local: pain, delayed healing, malodour	Enterococcus faecalis, Escherichia coli	Daytime - 6 Procedural - 9	No
4	Female (75)	**CHVI, DM, HT,** condition after varices surgery on the left lower leg	Venous leg ulcer at left lower leg	antiseptic dressing with silver nanoparticles	Local: pain, delayed healing	Staphylococcus aureus	Daytime - 5 Procedural - 7	No
5	Male (59)	Repeated diabetic gangrene, repeated amputation of fingers on the right foot, diabetic neuropathy DM, HT, obesity (BMI 32)	**Diabetic foot ulcer at right foot**	Iodinated povidone solution Antibiotic treatment – Gentamicin gel (local application)	Local: pain, exudate, delayed healing, malodour	Proteus mirabilis, Staphylococcus aureus, Acinetobacter baumanii	Daytime - 1 Procedural - 1	No
6	Male (54)	CHVI, HT, DM, hyperlipidemia, hyperuricemia	Venous leg ulcer at right lower leg	Iodinated povidone solution	Local: pain, delayed healing	Enterococcus faecalis	Daytime - 6 Procedural - 8	No
7	Male (52)	CHVI, DM, HT hyperlipidemia, hyperuricemia, morbid obesity (BMI 45), **repeated venous lower ulcers**	Bilateral venous leg ulcer	Iodinated povidone solution	Local: pain, delayed healing	Proteus mirabilis, Staphylococcus aureus	Daytime - 5 Procedural - 8	No
8	Female (51)	**DM, HT** morbid obesity (BMI 45), repeated wounds on the right foot of diabetic foot syndrome	**Diabetic foot ulcer at left foot**	Iodinated povidone solution	Local: pain, delayed healing	Proteus mirabilis, Staphylococcus aureus, Enterococcus faecalis	Daytime - 5 Procedural - 8	No
9	Male (49)	**DM, HT,** hyperlipidemia,	Venous leg ulcer at right lower leg	antiseptic dressing with silver nanoparticles	Local: pain, delayed healing, malodour	Enterococcus faecalis	Daytime - 5 Procedural - 8	No

	**condition after thrombosis of the right lower leg**

CHVI ***- Chronic venous insufficiency; DM – diabetes mellitus type 2; HT – hypertension; VAS –*** *visual analogue scale*
